# Antiviral activity of shikonin ester derivative PMM-034 against enterovirus 71 *in vitro*

**DOI:** 10.1590/1414-431X20176586

**Published:** 2017-08-17

**Authors:** Y. Zhang, H. Han, L. Sun, H. Qiu, H. Lin, L. Yu, W. Zhu, J. Qi, R. Yang, Y. Pang, X. Wang, G. Lu, Y. Yang

**Affiliations:** 1 State Key Laboratory of Pharmaceutical Biotechnology, NJU-NJFU Joint Institute of Plant Molecular Biology, Nanjing University, Nanjing, China; 2 Suzhou Industrial Park Center for Disease Control and Prevention, Suzhou, China; 3 College of Agriculture, Nanjing Agricultural University, Nanjing, China

**Keywords:** EV71, VP1, Shikonin ester derivatives PMM-034, Rhabdomyosarcoma cells, NF-κB

## Abstract

Human enterovirus 71 (EV71) is the major causative agent of hand, foot, and mouth disease (HFMD), particularly in infants and children below 4 years of age. Shikonin is a bioactive compound with anti-inflammatory, antiviral, and antibacterial activities derived from the roots of the Chinese medicinal herb *Lithospermum erythrorhizon*. This study aimed to examine the antiviral activity of PMM-034, a shikonin ester derivative, against EV71 in rhabdomyosarcoma (RD) cells. Cytotoxicity of PMM-034 on RD cells was determined using WST-1 assay. Dose- and time-dependent effects of PMM-034 on EV71 replication in RD cells were determined using plaque reduction assay. mRNA expression levels of EV71/VP1 and pro-inflammatory cytokines (IL-1β, IL-6, IL-8, and TNF-α) were determined by real-time RT-PCR, and EV71/VP1 and phospho-p65 protein expressions were determined by western blot analysis. PMM-034 exhibited only weak cytotoxicity against RD cells. However, PMM-034 exhibited significant antiviral activity against EV71 in RD cells with 50% inhibitory concentration of 2.31 μg/mL. The VP1 mRNA and protein levels were significantly reduced in cells treated with PMM-034. Furthermore, relative mRNA expression levels of IL-1β, IL-6, IL-8, and TNF-α significantly decreased in the cells treated with PMM-034, while the phospho-p65 protein expression was also significantly lower in the treated cells. These results indicated that PMM-034 suppressed the expressions of pro-inflammatory cytokines in RD cells, exhibiting antiviral activity against EV71, as evidenced by the reduced VP1 mRNA and protein levels in PMM-034-treated cells. Thus, PMM-034 is a promising candidate for further development as an EV71 inhibitor.

## Introduction

Hand, foot, and mouth disease (HFMD) is a common viral infection that commonly affects children below the age of 4 years (1). Human enterovirus 71 (EV71) belongs to the *Enterovirus* genus of the family *Picornaviridae* and causes sporadic outbreaks of HFMD. EV71 infection targets the central nervous system (CNS) (2). Once EV71 infects the CNS, a patient can die rapidly from severe complications including encephalitis and pulmonary edema (3). Recently, several consecutive EV71 epidemics have been reported in the Asia-Pacific region and no effective treatment for this infection exists. Selection of resistant viruses after treatment with antiviral drugs is inevitable (4), and while resistant viral strains have increased in recent years, only a few antiviral drugs have been approved for clinical use (5). The pace of pathogen resistance has exceeded the development of new antiviral drugs. Therefore, there is an urgent need to develop new effective antiviral compounds (4) to meet the growing need and combat resistance to existing antiviral drugs.

Signal transduction pathways are essential for normal viral immunity and for virus replication. Viral infections induce the production of pro-inflammatory cytokines and chemokines. A previous study has shown that excessive pro-inflammatory cytokine and chemokine responses were induced in human monocyte-derived macrophages, and interleukin (IL)-1β, IL-6, IL-8, and TNF-α levels, but not IFN-α and γ levels, were increased in EV71-infected patients (6). The NF-κB complex is activated in response to viral and bacterial infections, and the binding of the virion to its receptor can trigger membrane-proximal signaling cascades to activate NF-κB (7). Sometimes, viral products such as dsRNA and viral proteins can trigger NF-κB activation (8).

**Figure 1.**
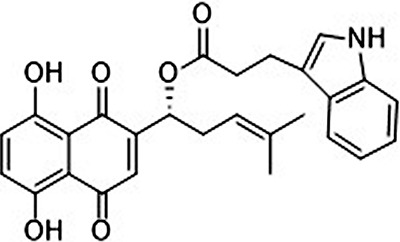
Chemical structure of PMM-034.

Several herbal prescriptions of traditional Chinese medicine (TCM) have been used to manage endemic infections due to their antiviral activity, for example, directly inhibiting or inactivating viruses, or improving the host immune system (9–11). Historically, root extracts obtained from the Chinese medicinal herb *L. erythrorhizon* have been used to treat burns, inflammation, trauma, and ulcers (12). Several studies have demonstrated that *L. erythrorhizon* has strong inhibitory activity towards neuraminidase and exhibits antiviral effects [i.e., against influenza A (H1N1) virus] by decreasing the viral reproduction and cytopathic effects (CPE) on virus-infected cells (13). Shikonin is an important naphthoquinone natural product derived from the roots of *L. erythrorhizon* (14). Shikonin exhibits a broad range of pharmacological activities including anti-oxidant, anti-cancer, anti-inflammatory and antibacterial effects (15,16). Shikonin may also exert antiviral activity, and it has been found to exhibit anti-human immunodeficiency virus-1 (HIV-1) and adenovirus activities (7,17). Recent research on shikonin has shifted to its derivatives (18), of which PMM-034 is one type (Figure 1). However, the antiviral effect of PMM-034 on EV71 infection remains unclear.

Here, we evaluated the extent to which PMM-034 could inhibit EV71 replication, and then assessed its effect on EV71/VP1 mRNA and protein expressions in rhabdomyosarcoma (RD) cells *in vitro*. Furthermore, we explored the stage in the replication cycle at which PMM-034 inhibits EV71 and assessed the anti-inflammatory activity of PMM-034 on EV71-induced inflammation *in vitro*.

## Material and Methods

### Cells and viruses

RD cells were purchased from the American Type Culture Collection (USA) and cultured in Dulbecco's modified Eagle's medium (DMEM; Gibco, USA) with 10% fetal bovine serum (FBS; Gibco). The cell culture plates were incubated in a humidified incubator containing 5% CO_2_ at 37°C.

EV71 was provided by the Suzhou Centre for Disease Prevention and Control (China). Finally, virus particles were amplified using RD cells, and the virus titer was determined by a plaque reduction assay using RD cells.

### Compounds

Shikonin ester derivatives were synthesized by our lab (19–21), and PMM-034 was dissolved in dimethyl sulfoxide (DMSO) for *in vitro* studies.

### Cytotoxicity assay

Cytotoxicity of PMM-034 against RD cells was determined using cell viability assay in 96-well plates with cells cultured to 80–90% confluence (approximately 5×104 cells/well). The cells were treated with various concentrations of PMM-034 dissolved in DMSO (0, 2.5, 5, 10, and 20 μg/mL) for 48 h, and then cell viability was assayed using WST-1 (Beyotime, China). Briefly, 10 μL of WST-1 solution was added to the cells and the suspension was incubated at 37°C for 2 h. Medium without cells was used as the blank control. Absorbance was measured at a wavelength of 450 nm using an AMR-100 Microplate Reader (ThermoRui-OS, USA). Cell viability was calculated using the following formula: Cell viability (%) = (absorbance (Ab)experimental group – Abblank control) / (Abnegative control – Abblank control) × 100%. The half-maximal cytotoxic concentration (CC50) was defined as the concentration that reduced the Ab of PMM-034-treated cells to 50% the Ab of negative control (untreated cells) (22).

### Plaque reduction assay

RD cells (3×105 cells/well) were seeded into 6-well plates and cultured to 80% confluence. After EV71 [80 plaque-forming units (pfu)/well] were incubated with RD cells to allow absorption for one hour at 37°C, the culture supernatants were replaced with fresh DMEM containing 1–3% FBS and 1.5% methyl cellulose, and then cultured for an additional 96 h at 37°C in the presence of 5% CO2. The cells were then washed with phosphate-buffered saline, fixed with 4% paraformaldehyde, stained with 5% crystal violet for 15 min, and then washed in running water. The plaques were counted under an inverted microscope (Nikon, TS100, Japan), and the virus titer (pfu/mL) was determined. Inhibition rate (%) was calculated as follows: [(mean number of plaques in the untreated group) – (mean number of plaques in the experimental group)] / (mean number of plaques in the untreated group) × 100 (23).

*Time-dependent effect of PMM-034 on EV71 replication in RD cells*. RD cells (3×10^5^ cells/well) were seeded and incubated in the wells of 6-well plates. RD cell monolayers were infected with EV71 at 80 pfu/well. After incubation for 1 h to facilitate absorption, culture supernatants were replaced with fresh DMEM containing 1–3% FBS. Then, 10 μg/mL PMM-034 was added to the cells at 0, 4, 8, 12, 16, and 24 h post-infection. Cell supernatants were collected for the viral plaque reduction assay.

*Dose-dependent effect of PMM-034 on EV71 replication in RD cells*. RD cells (3×10^5^ cells/well) were plated into 6-well culture plates and incubated for 24 h. The medium was then removed and cells were infected with EV71 at 80 pfu/well. After virus absorption for 1 h, the medium was aspirated from the wells to remove unabsorbed virus, and the cells were washed thrice with serum-free DMEM, and treated with different PMM-034 concentrations to test for antiviral activity. Finally, EV71-infected cells and culture supernatants were collected 48 h after infection for the plaque reduction assay.

### Quantitative real-time reverse transcription (RT)-PCR

Total RNA was extracted using Trizol (Invitrogen, USA) according to the manufacturer's instructions. The RNA purity and concentration were determined by spectroscopy [Ab260/Ab280] to be 1.8–2.0. RNA was reverse transcribed using a Reverse Transcription System (Promega, USA). Primers for *IL-1*, *IL-6*, *IL-8*, *TNF-α*, *VP1* and *GAPDH* were designed using Beacon Designer 7.0 and synthesized by Sangon Biotech Company (China; Table 1). Specific mRNAs were quantified by real-time PCR using Fast SYBR Green Master Mix (Invitrogen) in a Bio Rad CFX96 Real-Time System (Bio Rad, USA). Real-time PCR reactions were performed in triplicate in 96-well plates under the following conditions; 15 s at 95°C followed by 40 cycles of 15 s at 95°C, 30 s at 65°C, and 30 s at 72°C. GAPDH was used as the internal control. The relative expression levels of the genes were compared with those of GAPDH by the 2^–ΔΔCt^ method.

**Table 1.** Primers used for real-time PCR.

Gene	Sequence (5′-3′)
VP1	F: TGGCAGATGTGATTGAGAG
	R: GGCTTGAAGTGCTGGTA
IL-1β	F: ATGATGGCTTATTACAGTGGCAA
	R: GTCGGAGATTCGTAGCTGGA
IL-6	F: AGGAGACTTGCCTGGTGAAA
	R: CAGGGGTGGTTATTGCATCT
IL-8	F: TTGGCAGCCTTCCTGATTTC
	R: TCTTTAGCACTCCTTGGCAAAAC
TNF-α	F: GAGGCCAAGCCCTGGTATG
	R: CGGGCCGATTGATCTCAGC
GAPDH	F: GCACCGTCAAGGCTGAGAAC
	R: TGGTGAAGACGCCAGTGGA

F: forward; R: reverse.

### Western blot analysis

Total protein was extracted using radio-immunoprecipitation assay plus phenylmethylsulfonyl fluoride (Solarbio, China) according to the manufacturer's instructions. Protein concentration was determined by the BCA Protein Assay Kit (Thermo, USA). Equal amounts of protein (60 μg) were subjected to sodium dodecyl sulfate-polyacrylamide gel electrophoresis using 10% gels. Proteins were transferred to nitrocellulose membrane (GE Healthcare, USA). After the transfer, the membranes were blocked with 5% (w/v) nonfat dry milk in TBS-T (10 mM Tris-HCl, pH 8.0, 150 mM NaCl containing 0.1% v/v Tween-20) for at least 1 h. The membranes were then incubated with the following primary antibodies: Anti-Norovirus capsid protein VP1 antibodies (ab92976, 1:500; Abcam, UK); phospho-NF-κB/p65 (S536) rabbit antibodies (3013, 1:1000; CST, USA); β-actin mouse monoclonal antibodies (A1978, 1:3000; Sigma Aldrich, USA) in blocking buffer overnight at 4°C. After washing thrice with TBST, membranes were incubated with secondary antibodies for 30 min and detected using an ECL PLUS detection kit (Thermo).

### Statistical analysis

Statistical analyses were conducted using SPSS 17.0 software. Data are reported as means±SD from at least three independent experiments performed in triplicate. Statistical significance was evaluated by the Student's *t*-test. P<0.05 was considered to be statistically significant.

## Results

### Cytotoxicity of PMM-034 on RD cells

**Figure 2.**
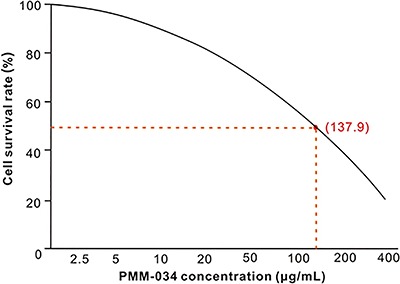
Cytotoxicity of shikonin ester derivative PMM-034 on rhabdomyosarcoma cell viability. PMM-034 was serially diluted to 0, 2.5, 5, 10, 20, 50, 100, 200, and 400 μg/mL in DMEM containing 2.5% FBS. Subsequently, the cytotoxicity of PMM-034 on RD cells was determined by the WST-1 assay. Data are reported as means ± standard deviation (SD) from at least three independent experiments. The red dot indicates the PMM-034 concentration under 50% cell survival rate.

The results of the WST-1 assay revealed that PMM-034 did not significantly affect cell viability at concentrations of ≤100 μg/mL, and its CC_50_ was 137.9 μg/mL. This finding suggested that PMM-034 was only weakly cytotoxic to RD cells (Figure 2).

### Dose- and time-dependent effects of PMM-034 on EV71 replication in RD cells

**Figure 3.**
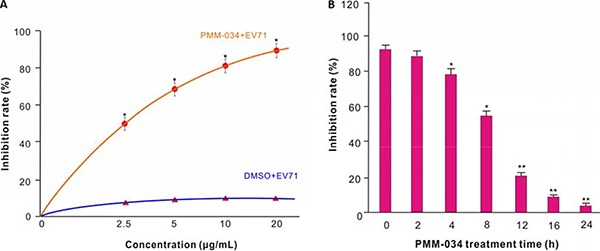
Dose- and time-dependent effects of PMM-034 on human enterovirus 71 (EV71) replication in rhabdomyosarcoma (RD) cells. *A*, RD cells were infected with EV71 for 1 h, then treated with PMM-034 or DMSO for 23 h. Infected cells and culture supernatants were collected 48 h post-infection. *B*, RD cells were infected with EV71 for 1 h, and 10 μg/mL PMM-034 was added at 0, 2, 4, 8, 12, 16, and 24 h after infection. Virus titers were measured by the plaque reduction assay. Data are reported as means±SD from at least 3 independent experiments. *P<0.05; **P<0.01, Student’s t-test.

The plaque reduction assay using culture supernatants showed inhibition rates of 54.9±1.4, 67.9±1.7, 79.8±3.1, and 91.3±1.9%, respectively, for the tested concentrations. The 50% inhibitory concentration (IC_50_) of PMM-034 for EV71 replication was 2.31 μg/mL. EV71 replication was not inhibited in EV71-infected RD cells treated with the same concentration of DMSO (Figure 3A). The result showed that PMM-034 exhibited strong antiviral activity against EV71 in a dose-dependent manner.

A time course assay was performed to explore the stages of the EV71 viral replication cycle that were affected by PMM-034. EV71 replication in RD cells was suppressed from 0–8 h, whereas only a partial inhibitory effect was observed at 12–24 h (Figure 3B). Therefore, anti-EV71 activity of PMM-034 was most prominent when it was applied early in the EV71 replication cycle.

### PMM-034 reduced the mRNA and protein expressions of EV71 VP1

**Figure 4.**
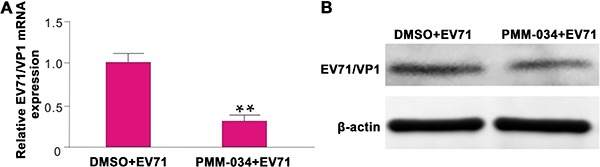
Effects of PMM-034 on human enterovirus 71 (EV71/VP1) mRNA and protein expressions in rhabdomyosarcoma (RD) cells. RD cells were infected with EV71 for 1 h and then treated with 10 μg/mL PMM-034 or DMSO for 23 h. *A*, The EV71/VP1 mRNA level was detected by real-time RT-PCR. GAPDH was used as the internal control. Data are reported as means±SD of at least 3 independent experiments, **P<0.01, Student’s t-test. *B*, Expression levels of EV71/VP1 protein were detected by western blotting. β-actin was used as the internal control.

To study how PMM-034 affected the mRNA and protein levels of EV71/VP1, total RNA was extracted from EV71-infected cells treated with or without PMM-034 for 24 h. The results of the qRT-PCR revealed that EV71 VP1 mRNA level was significantly reduced in the PMM-034-treated cells (Figure 4A). Consistent with these results, PMM-034 decreased the expression of VP1 protein (Figure 4B).

### PMM-034 reduced the severity of EV71-induced inflammation

**Figure 5.**
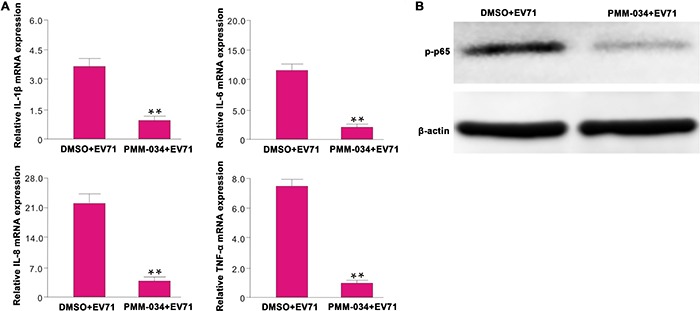
Effects of PMM-034 on the mRNA levels of pro-inflammatory cytokines and phospho-p65 protein expression in rhabdomyosarcoma (RD) cells. RD cells were infected with human enterovirus 71 (EV71) for 1 h, then treated with 10 μg/mL PMM-034 or DMSO for 23 h. *A*, mRNA levels of IL-1β, IL-6, IL-8, and TNF-α were detected by real-time RT-PCR. GAPDH was used as the internal control. Data are reported as means±SD of at least 3 independent experiments. **P<0.01, Student’s t-test. *B*, Phospho-p65 protein expression was detected by western blotting. β-actin was used as the internal control.

The TNF-α, IL-1β, IL-6, and IL-8 expression levels were significantly lower in EV71-infected RD cells treated with PMM-034 compared to DMSO-treated EV71-infected cells (Figure 5A).

Furthermore, PMM-034 decreased the phospho-p65 protein levels, which might account for the reduction of pro-inflammatory cytokines in EV71-infected RD cells treated with PMM-034 (Figure 5B).

## Discussion

Certain TCMs have shown antiviral activity (24), and often function via multiple mechanisms of action and are associated with few side effects and no drug resistance (25). Lariciresinol-4-*O*-*β*-d-glucopyranoside, which is one of lignin compounds extracted from the root of *Isatis indigotica*, has been found to be effective against influenza A virus-induced CPE in MDCK cells. These studies also revealed that the lignin glycoside suppressed H1N1-induced expression of the proinflammatory molecules IL-6, TNF-α, IL-8, MCP- 1, IP-10, and IFN-α (26). Baicalin, a flavonoid compound extracted from *Scutellaria* roots, also exhibits potent antiviral effects on EV71 infection through inhibition of EV71/3D polymerase expression and Fas/FasL signaling pathways (27). Shikonin also possesses anti-AdV3 capabilities and the potential antiviral mechanism might involve inhibition of the degree of apoptosis and hexon protein expression of AdV (7). Recent research on shikonin has shifted to its derivatives, of which PMM-034 is one type. To date, it is still poorly understood whether shikonin can inhibit EV71 infection, let alone the antiviral activity of PMM-034 on EV71. In the present study, we found that ≤100 μg/mL PMM-034 was not cytotoxic to RD cells. We found that PMM-034 exhibited a strong antiviral effect against EV71, and could inhibit 54.9-91.3% of the virus in a concentration-dependent manner. Furthermore, the IC_50_ of this compound against EV71 was 2.31 μg/mL. PMM-034 significantly suppressed EV71 replication in RD cells at 0–8 h after infection, but weakly inhibited replication at 12–24 h after infection. This indicates that the antiviral effects of PMM-034 occurred mainly at the early stage of EV71 infection.

The EV71 genome consists of a positive-sense single-stranded 7400-bp RNA (28). Upon infection, the internal ribosome entry site element in the 5′-untranslated region drives the translation of the viral processor polyprotein (29). This polyprotein, which is encoded as NH_2_-VP4-VP2-VP3-VP1-2A-2B-2C-3A-3B-3C-3D-COOH, is then proteolytically cleaved by viral 2A and 3C proteases to form a range of viral structural proteins (VP1, VP2, VP3, and VP4) and 7 non-structural viral proteins (2A, 2B, 2C, 3A, 3B, 3C, and 3D) (30). The first three viral proteins (VP1–VP3) are present on the outer surface of the virus, and the shorter VP4 is located completely on the inner surface of the capsid. The capsid proteins initiate infection by binding to a receptor on the host membrane (31). VP1 is the most external and immunodominant of the picornavirus capsid proteins, and the VP1 sequence may be applied to classify enteroviruses and to analyze the phylogenetic relationships among human enteroviruses (32). VP1 has often been used for EV71 molecular genotyping and epidemiological monitoring (33). Here, we found that VP1 protein expression was significantly blocked by PMM-034 at the early stage of EV71 infection. Furthermore, EV71 mRNA abundance was also reduced after treatment with PMM-034, which was consistent with the results of the western blot analysis.

Previous studies have shown that the severity of clinical manifestations associated with EV71 infection possibly depend on the host immune inflammatory response, including pro-inflammatory and anti-inflammatory cytokines and chemokine storms in the blood and cerebrospinal fluid (34). Systemic inflammation caused by EV71 infection further deteriorated CNS disease, resulting in disease progression to the critical illness stage (35). NLRP3 inflammasomes have been shown to play a crucial role in the pathogenesis of coxsackievirus B3-induced myocarditis (36). Infection with CBV3, an enterovirus of the Piconaviridae family, induced production of IL-1β in cardiac tissues of VMC mice, which was positively correlated with the severity of myocarditis (37). Excessive host immune responses may play a critical role in the course of CVB3-induced myocarditis (37). In addition, IAV-elicited NF-κB activity helps viral replication and spreading, and inhibition of IKK activity by the small molecule inhibitors BAY11-7085 and BAY11-7082 severely impaired IAV infection in human lung carcinoma cell lines (38). It is well known that active NF-κB is a transcription factor that induces hundreds of genes as part of an adjustment program to cope with the danger and stress signals leading to NF-κB activation. The target genes of NF-κB include regulators of inflammatory cytokines (e.g., IL-8) and cell survival, proliferation, and cell surface proteins (38). In this study, EV71 infection enhanced the expression of pro-inflammatory cytokines such as TNF-α, IL-1β, IL-6, and IL-8. We further showed that PMM-034 could suppress the phosphorylation of NF-κB/p65 induced by EV71 infection. Therefore, we propose that PMM-034 might reduce the severity of the inflammatory response involved in the inhibition of NF-κB activation. Researchers usually choose non-human primates like green monkeys, cynomolgus, rhesus, and mouse models to investigate the pathogenesis of EV71 infection. Arita and his group reported that neonatal mice are susceptible to EV71 infection, and the mice exhibited an age-dependent susceptibility to EV71 infection (39). Mice older than 14 days were resistant to infection with EV71 clinical isolates. Yu et al. reported that EV71 infected immunocompetent-ICR mice developed rear limb paralysis and neuropathologies in the brain stem and spinal cord before death. For 1 to 7 days, MP-26 M infections in BALB/c mice can lead to limb paralysis, and the viruses can be isolated from skeletal muscle, blood, brains, livers, spleens and hearts (40). In the next step, we may also use neonatal mice as animal models to further investigate the inhibition effect of PMM-034 on EV71 infection and the underlying mechanism.

In this study, we found that PMM-034 could effectively suppress the expression of pro-inflammatory cytokines in EV71-infected RD cells, and exhibit antiviral activity against EV71, as evidenced by the reduced VP1 mRNA and protein levels in PMM-034-treated cells. Additionally, PMM-034 was not found to exert significant cytotoxicity against RD cells, making it a promising candidate for further development as an EV71 inhibitor.
